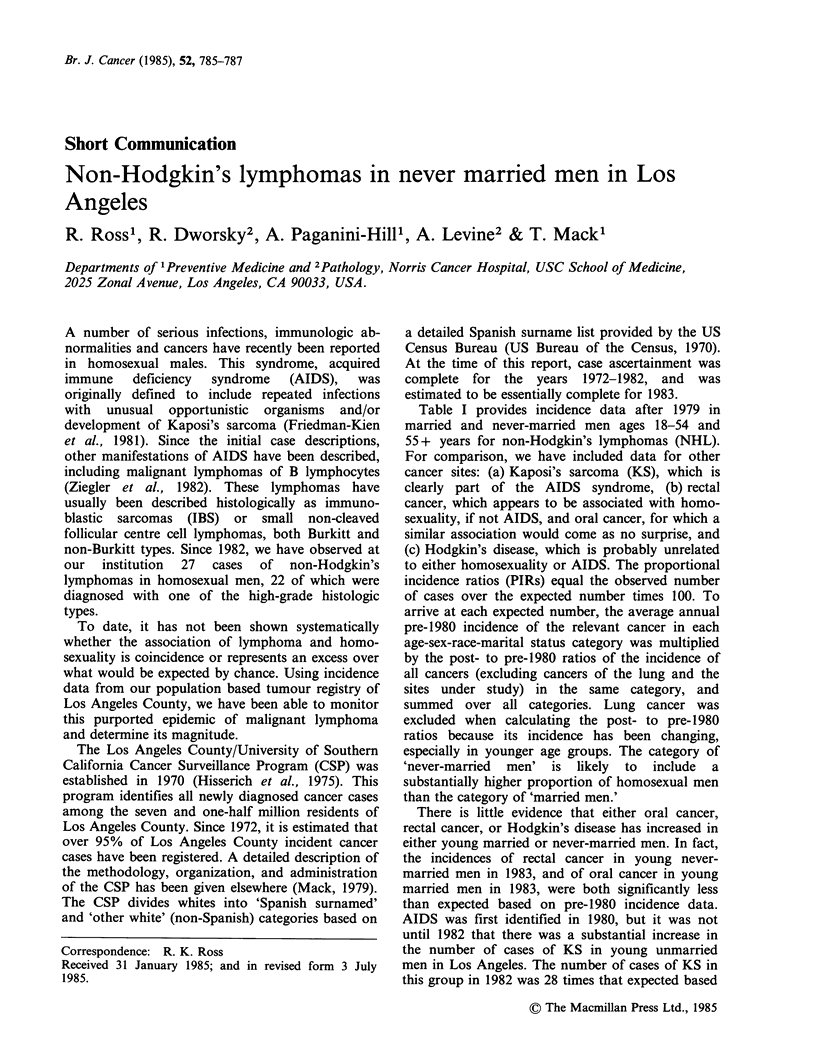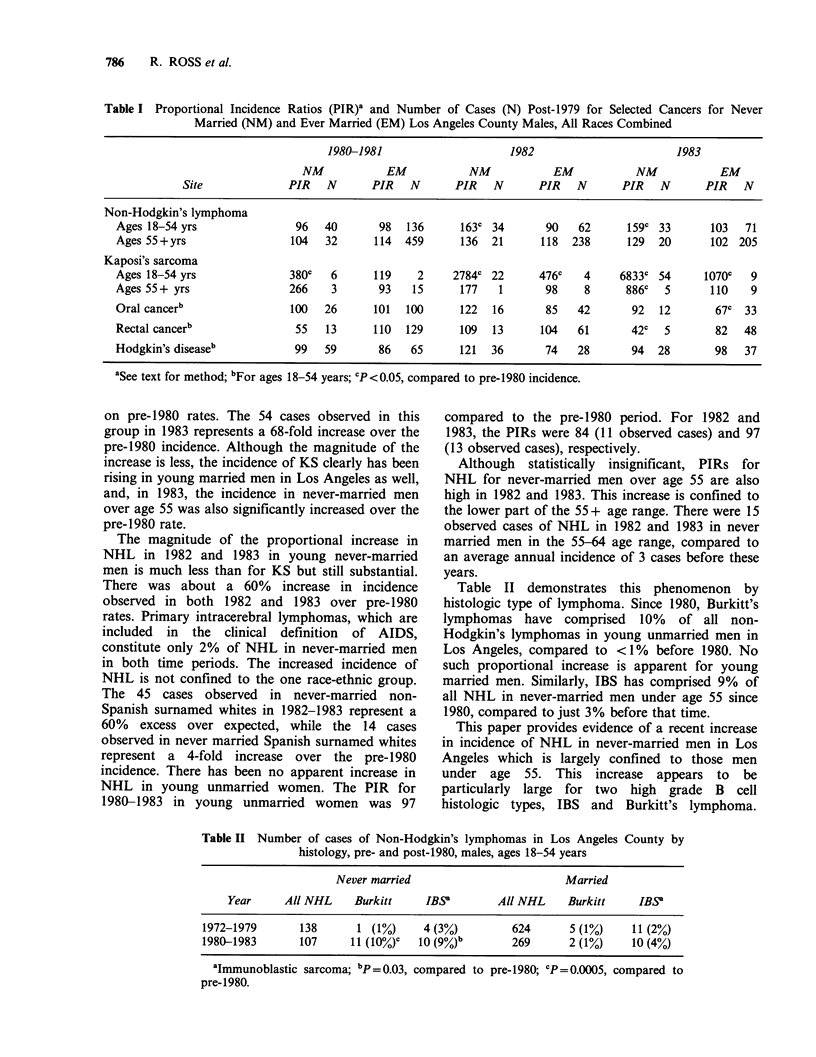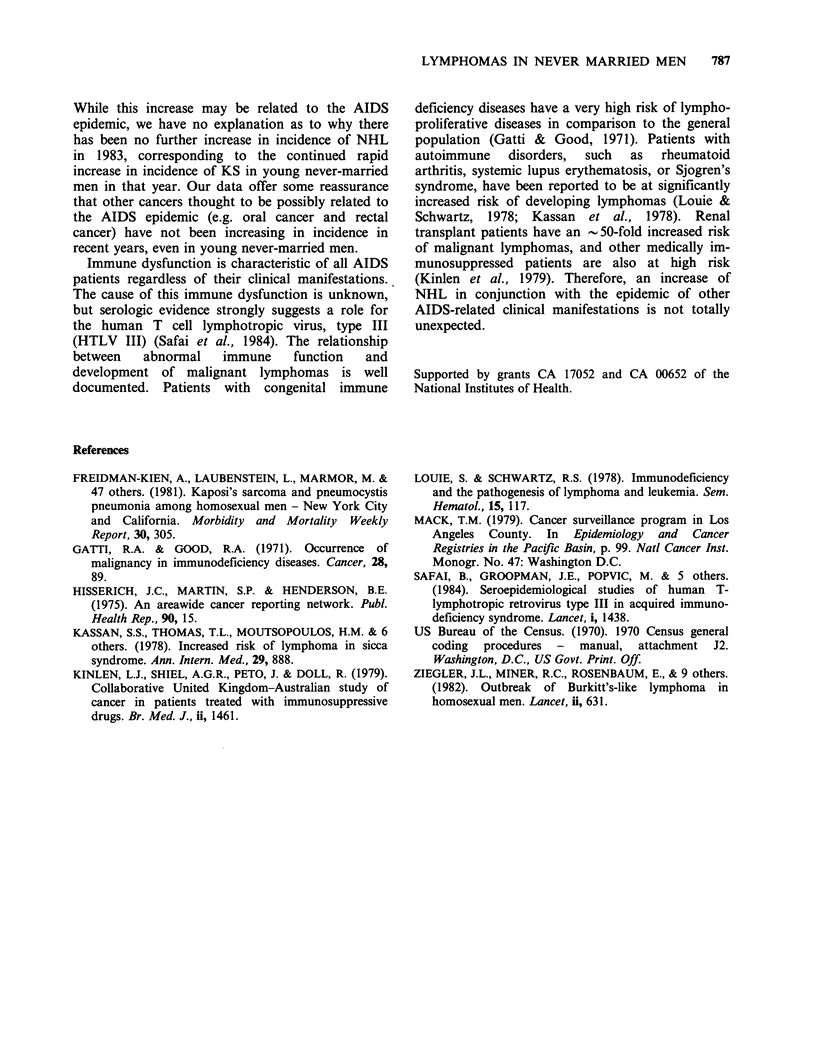# Non-Hodgkin's lymphomas in never married men in Los Angeles.

**DOI:** 10.1038/bjc.1985.258

**Published:** 1985-11

**Authors:** R. Ross, R. Dworsky, A. Paganini-Hill, A. Levine, T. Mack


*Br. J. Cancer* (1985), **52**, 785–787

**Short Communication**

### Non-Hodgkin's lymphomas in never married men in Los Angeles

R. Ross[1], R. Dworsky[2], A. Paganini-Hill[1], A. Levine[2] & T. Mack[1]

*Departments of [1]Preventive Medicine and [2]Pathology, Norris Cancer Hospital, USC School of Medicine, 2025 Zonal Avenue, Los Angeles, CA 90033, USA.*

A number of serious infections, immunologic abnormalities and cancers have recently been reported in homosexual males. This syndrome, acquired immune deficiency syndrome (AIDS), was originally defined to include repeated infections with unusual opportunistic organisms and/or development of Kaposi's sarcoma (Friedman-Kien *et al.,* 1981). Since the initial case descriptions, other manifestations of AIDS have been described, including malignant lymphomas of B lymphocytes (Ziegler *et al.,* 1982). These lymphomas have usually been described histologically as immunoblastic sarcomas (IBS) or small non-cleaved follicular centre cell lymphomas, both Burkitt and non-Burkitt types. Since 1982, we have observed at our institution 27 cases of non-Hodgkin's lymphomas in homosexual men, 22 of which were diagnosed with one of the high-grade histologic types.

To date, it has not been shown systematically whether the association of lymphoma and homosexuality is coincidence or represents an excess over what would be expected by chance. Using incidence data from our population based tumour registry of Los Angeles County, we have been able to monitor this purported epidemic of malignant lymphoma and determine its magnitude.

The Los Angeles County/University of Southern California Cancer Surveillance Program (CSP) was established in 1970 (Hisserich *et al.,* 1975). This program identifies all newly diagnosed cancer cases among the seven and one-half million residents of Los Angeles County. Since 1972, it is estimated that over 95% of Los Angeles County incident cancer cases have been registered. A detailed description of the methodology, organization, and administration of the CSP has been given elsewhere (Mack, 1979). The CSP divides whites into 'Spanish surnamed' and 'other white' (non-Spanish) categories based on a detailed Spanish surname list provided by the US Census Bureau (US Bureau of the Census, 1970). At the time of this report, case ascertainment was complete for the years 1972–1982, and was estimated to be essentially complete for 1983.

Table I provides incidence data after 1979 in married and never-married men ages 18–54 and 55+ years for non-Hodgkin's lymphomas (NHL). For comparison, we have included data for other cancer sites: (a) Kaposi's sarcoma (KS), which is clearly part of the AIDS syndrome, (b) rectal cancer, which appears to be associated with homosexuality, if not AIDS, and oral cancer, for which a similar association would come as no surprise, and (c) Hodgkin's disease, which is probably unrelated to either homosexuality or AIDS. The proportional incidence ratios (PIRs) equal the observed number of cases over the expected number times 100. To arrive at each expected number, the average annual pre-1980 incidence of the relevant cancer in each age-sex-race-marital status category was multiplied by the post- to pre-1980 ratios of the incidence of all cancers (excluding cancers of the lung and the sites under study) in the same category, and summed over all categories. Lung cancer was excluded when calculating the post- to pre-1980 ratios because its incidence has been changing, especially in younger age groups. The category of 'never-married men' is likely to include a substantially higher proportion of homosexual men than the category of 'married men.'

There is little evidence that either oral cancer, rectal cancer, or Hodgkin's disease has increased in either young married or never-married men. In fact, the incidences of rectal cancer in young never-married men in 1983, and of oral cancer in young married men in 1983, were both significantly less than expected based on pre-1980 incidence data. AIDS was first identified in 1980, but it was not until 1982 that there was a substantial increase in the number of cases of KS in young unmarried men in Los Angeles. The number of cases of KS in this group in 1982 was 28 times that expected based

Correspondence: R. K. Ross
Received 31 January 1985; and in revised form 3 July 1985.

© The Macmillan Press Ltd., 1985

**Table I** Proportional Incidence Ratios (PIR)[a] and Number of Cases (N) Post-1979 for Selected Cancers for Never Married (NM) and Ever Married (EM) Los Angeles County Males, All Races Combined

| | *1980–1981* | | | | *1982* | | | | *1983* | | | |
|---|---|---|---|---|---|---|---|---|---|---|---|---|
| | *NM* | | *EM* | | *NM* | | *EM* | | *NM* | | *EM* | |
| *Site* | *PIR* | *N* | *PIR* | *N* | *PIR* | *N* | *PIR* | *N* | *PIR* | *N* | *PIR* | *N* |
| Non-Hodgkin's lymphoma | | | | | | | | | | | | |
| Ages 18–54 yrs | 96 | 40 | 98 | 136 | 163[c] | 34 | 90 | 62 | 159[c] | 33 | 103 | 71 |
| Ages 55+yrs | 104 | 32 | 114 | 459 | 136 | 21 | 118 | 238 | 129 | 20 | 102 | 205 |
| Kaposi's sarcoma | | | | | | | | | | | | |
| Ages 18–54 yrs | 380[c] | 6 | 119 | 2 | 2784[c] | 22 | 476[c] | 4 | 6833[c] | 54 | 1070[c] | 9 |
| Ages 55+ yrs | 266 | 3 | 93 | 15 | 177 | 1 | 98 | 8 | 886[c] | 5 | 110 | 9 |
| Oral cancer[b] | 100 | 26 | 101 | 100 | 122 | 16 | 85 | 42 | 92 | 12 | 67[c] | 33 |
| Rectal cancer[b] | 55 | 13 | 110 | 129 | 109 | 13 | 104 | 61 | 42[c] | 5 | 82 | 48 |
| Hodgkin's disease[b] | 99 | 59 | 86 | 65 | 121 | 36 | 74 | 28 | 94 | 28 | 98 | 37 |

[a]See text for method; [b]For ages 18–54 years; [c]$P<0.05$, compared to pre-1980 incidence.

on pre-1980 rates. The 54 cases observed in this group in 1983 represents a 68-fold increase over the pre-1980 incidence. Although the magnitude of the increase is less, the incidence of KS clearly has been rising in young married men in Los Angeles as well, and, in 1983, the incidence in never-married men over age 55 was also significantly increased over the pre-1980 rate.

The magnitude of the proportional increase in NHL in 1982 and 1983 in young never-married men is much less than for KS but still substantial. There was about a 60% increase in incidence observed in both 1982 and 1983 over pre-1980 rates. Primary intracerebral lymphomas, which are included in the clinical definition of AIDS, constitute only 2% of NHL in never-married men in both time periods. The increased incidence of NHL is not confined to the one race-ethnic group. The 45 cases observed in never-married non-Spanish surnamed whites in 1982–1983 represent a 60% excess over expected, while the 14 cases observed in never married Spanish surnamed whites represent a 4-fold increase over the pre-1980 incidence. There has been no apparent increase in NHL in young unmarried women. The PIR for 1980–1983 in young unmarried women was 97 compared to the pre-1980 period. For 1982 and 1983, the PIRs were 84 (11 observed cases) and 97 (13 observed cases), respectively.

Although statistically insignificant, PIRs for NHL for never-married men over age 55 are also high in 1982 and 1983. This increase is confined to the lower part of the 55+ age range. There were 15 observed cases of NHL in 1982 and 1983 in never married men in the 55–64 age range, compared to an average annual incidence of 3 cases before these years.

Table II demonstrates this phenomenon by histologic type of lymphoma. Since 1980, Burkitt's lymphomas have comprised 10% of all non-Hodgkin's lymphomas in young unmarried men in Los Angeles, compared to <1% before 1980. No such proportional increase is apparent for young married men. Similarly, IBS has comprised 9% of all NHL in never-married men under age 55 since 1980, compared to just 3% before that time.

This paper provides evidence of a recent increase in incidence of NHL in never-married men in Los Angeles which is largely confined to those men under age 55. This increase appears to be particularly large for two high grade B cell histologic types, IBS and Burkitt's lymphoma.

**Table II** Number of cases of Non-Hodgkin's lymphomas in Los Angeles County by histology, pre- and post-1980, males, ages 18–54 years

| | *Never married* | | | *Married* | | |
|---|---|---|---|---|---|---|
| *Year* | *All NHL* | *Burkitt* | *IBS*[a] | *All NHL* | *Burkitt* | *IBS*[a] |
| 1972–1979 | 138 | 1 (1%) | 4 (3%) | 624 | 5 (1%) | 11 (2%) |
| 1980–1983 | 107 | 11 (10%)[c] | 10 (9%)[b] | 269 | 2 (1%) | 10 (4%) |

[a]Immunoblastic sarcoma; [b]$P=0.03$, compared to pre-1980; [c]$P=0.0005$, compared to pre-1980.

While this increase may be related to the AIDS epidemic, we have no explanation as to why there has been no further increase in incidence of NHL in 1983, corresponding to the continued rapid increase in incidence of KS in young never-married men in that year. Our data offer some reassurance that other cancers thought to be possibly related to the AIDS epidemic (e.g. oral cancer and rectal cancer) have not been increasing in incidence in recent years, even in young never-married men.

Immune dysfunction is characteristic of all AIDS patients regardless of their clinical manifestations. The cause of this immune dysfunction is unknown, but serologic evidence strongly suggests a role for the human T cell lymphotropic virus, type III (HTLV III) (Safai *et al.*, 1984). The relationship between abnormal immune function and development of malignant lymphomas is well documented. Patients with congenital immune deficiency diseases have a very high risk of lymphoproliferative diseases in comparison to the general population (Gatti & Good, 1971). Patients with autoimmune disorders, such as rheumatoid arthritis, systemic lupus erythematosis, or Sjogren's syndrome, have been reported to be at significantly increased risk of developing lymphomas (Louie & Schwartz, 1978; Kassan *et al.*, 1978). Renal transplant patients have an ~50-fold increased risk of malignant lymphomas, and other medically immunosuppressed patients are also at high risk (Kinlen *et al.*, 1979). Therefore, an increase of NHL in conjunction with the epidemic of other AIDS-related clinical manifestations is not totally unexpected.

Supported by grants CA 17052 and CA 00652 of the National Institutes of Health.

**References**

FREIDMAN-KIEN, A., LAUBENSTEIN, L., MARMOR, M. & 47 others. (1981). Kaposi's sarcoma and pneumocystis pneumonia among homosexual men – New York City and California. *Morbidity and Mortality Weekly Report*, **30**, 305.

GATTI, R.A. & GOOD, R.A. (1971). Occurrence of malignancy in immunodeficiency diseases. *Cancer*, **28**, 89.

HISSERICH, J.C., MARTIN, S.P. & HENDERSON, B.E. (1975). An areawide cancer reporting network. *Publ. Health Rep.*, **90**, 15.

KASSAN, S.S., THOMAS, T.L., MOUTSOPOULOS, H.M. & 6 others. (1978). Increased risk of lymphoma in sicca syndrome. *Ann. Intern. Med.*, **29**, 888.

KINLEN, L.J., SHIEL, A.G.R., PETO, J. & DOLL, R. (1979). Collaborative United Kingdom–Australian study of cancer in patients treated with immunosuppressive drugs. *Br. Med. J.*, **ii**, 1461.

LOUIE, S. & SCHWARTZ, R.S. (1978). Immunodeficiency and the pathogenesis of lymphoma and leukemia. *Sem. Hematol.*, **15**, 117.

MACK, T.M. (1979). Cancer surveillance program in Los Angeles County. In *Epidemiology and Cancer Registries in the Pacific Basin*, p. 99. *Natl Cancer Inst.* Monogr. No. 47: Washington D.C.

SAFAI, B., GROOPMAN, J.E., POPVIC, M. & 5 others. (1984). Seroepidemiological studies of human T-lymphotropic retrovirus type III in acquired immunodeficiency syndrome. *Lancet*, **i**, 1438.

US Bureau of the Census. (1970). 1970 Census general coding procedures – manual, attachment J2. *Washington, D.C., US Govt. Print. Off.*

ZIEGLER, J.L., MINER, R.C., ROSENBAUM, E., & 9 others. (1982). Outbreak of Burkitt's-like lymphoma in homosexual men. *Lancet*, **ii**, 631.